# The In Vivo Chondrotoxicity of Single Intra-articular Injection of Local Anesthetic in Rat Cartilage

**DOI:** 10.7759/cureus.53103

**Published:** 2024-01-28

**Authors:** Ioannis Vrachnis, John Gliatis, Dionysios Papachristou, Sofia Sourouni, Antonis Kouzelis, Andreas Panagopoulos, Minos Tyllianakis

**Affiliations:** 1 Department of Orthopaedics, Patras University Hospital, Patras, GRC; 2 Department of Orthopaedics, School of Medicine, University of Patras, Patras, GRC; 3 Department of Anatomy, School of Medicine, University of Patras, Patras, GRC; 4 Department of Radiology, Patras University Hospital, Patras, GRC

**Keywords:** ropivacaine, lidocaine, local anesthetic, chondrotoxicity, cartilage

## Abstract

Introduction

A constant infusion of local anesthetics through pain pumps has been shown to cause chondrolysis. However, there is no general consensus regarding the safety of a single intra-articular injection of local anesthetics. In this experimental study, we examined the rat cartilage for possible histological effects after a single intra-articular administration of lidocaine or ropivacaine.

Material and methods

Thirty-two male Sprague-Dawley rats, weighing 250-300 grams, were divided into two groups of 16 each. We injected 0.1 ml of either lidocaine 2% (20 mg/ml) or ropivacaine 0.75% (7.5 mg/ml) into the left knee of the rats. The right knee in both groups was used as a control, and an equal amount of normal saline was injected. Each group was further divided into subgroups of four, which were euthanized after one, seven, 21, and 60 days after the initial injection. Knees were excised and prepared for histopathological analysis. A modified version of the Mankin score was used for cartilage damage evaluation.

Results

No difference regarding cartilage damage was detected after the examination under light microscopy between lidocaine, ropivacaine, and placebo in all specimens. Time elapsed since the initial injection did not affect the results at any time point.

Conclusion

A single intra-articular injection of local anesthetic did not induce any histological changes in the rat cartilage. Further research is needed to demonstrate the safety of humans.

## Introduction

Intra-articular use of local anesthetics is very common in clinical practice. Simple arthroscopic procedures may be performed solely under local anesthesia, as well as infiltration of portals after arthroscopic procedures for postoperative pain management or intra-articular knee injections combined with steroids or hyaluronic acid [1]. Local anesthesia reduces the complication rate and results in superior perioperative pain management compared to other anesthesia methods [2] without affecting patient satisfaction [3].

Early experimental data did not support the induction of toxic effects on cartilage from the use of local anesthetics [4]. The clinical evidence of cartilage toxicity was established when the use of pain pumps became widespread. An increased number of cases of chondrolysis related to the use of continuous infusion pain pumps was observed [5]. The vast majority of the cases (almost 90%) have been reported to affect the glenohumeral joint, which is considered to be the most vulnerable [5]. Reports of chondrolysis affecting the knee joint have also been published [6]. There seems to be a positive correlation between the concentration of the anesthetic and the frequency of the side effects [7].

Following clinical observation, a number of in vitro and in vivo studies have been conducted. Cartilage inflammation [8], impaired mitochondrial function, chondrocyte apoptosis [9], and alteration in the expression of genes related to collagen and aggrecan formation [10] have been reported. The toxicity of the different local anesthetics seems to vary. Lidocaine is considered to cause the most toxic effects, while ropivacaine seems to induce less damage [11-12]. However, some studies have not demonstrated severe damage to the cartilage, especially when a single dose of anesthetic was used [13-14]. Thus, potential cartilage toxicity arising from a single intra-articular dose of local anesthetic remains controversial.

In this experimental study, we aimed to test the safety of a single intra-articular injection of two widely used local anesthetics, lidocaine and ropivacaine, on normal rat cartilage in vivo. Our main hypothesis is that no toxic cartilage effects are expected after a single intra-articular local anesthetic administration. If cartilage toxicity is observed, we expect less or minimal damage from ropivacaine. The cartilage specimens were examined at variable time intervals after the initial injection. In order to verify our technique regarding proper intra-articular administration, the injections took place under ultrasound (US) guidance.

## Materials and methods

**Figure 1:**
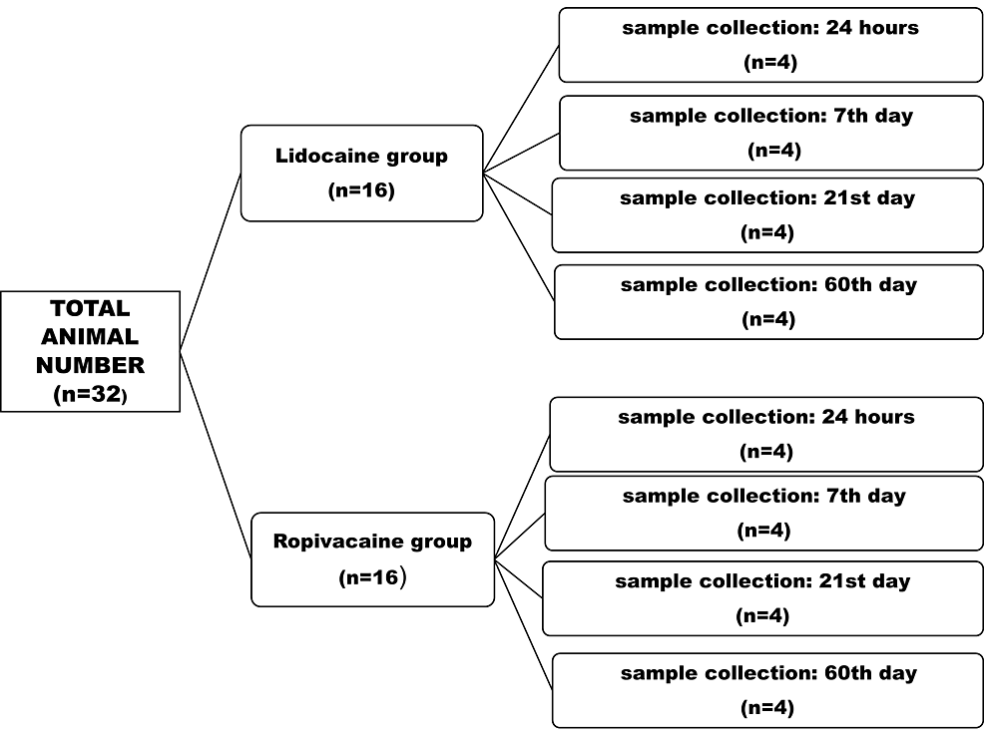
Experimental protocol The initial number of 32 rats was divided in order to test two different substances (lidocaine and ropivacaine). Each rat group was further divided into groups of four rats, which were sacrificed at different time intervals after the intra-articular injection.

After approval by the local bioethical committee (Region of Western Greece, Veterinary Department ΑΠ ΠΔΕ/ΔΚ/11438/65), 32 male Sprague-Dawley rats, weighting 250-300 grams each, were used for the purposes of our study. They were divided into two groups of 16 (Figure 1). The first group received lidocaine 20 mg/ml (Xylocaine 2%) and the second group received ropivacaine hydrochloride monohydrate 7.5 mg/ml (Naropeine 0.75%). Normal saline solution was injected into the contralateral knee in both groups. The volume of either anesthetic injected into the rat’s knee was 0.1 ml. This volume is considered to be equivalent to a volume infused in the human knee in arthroscopic procedures under local anesthesia [15-17].

The total number of animals was obtained according to Mead's resource equation [18-19] an alternative to the power analysis method used in animal studies. Each group of 16 rats was further divided into subgroups of four (Figure 1). The rats belonging to the first subgroup were euthanized one day after the intra-articular injection of the local anesthetic. Those belonging to the second, third, and fourth subgroups were euthanized after seven, 21, and 60 days of the intra-articular injection, respectively.

**Figure 2:**
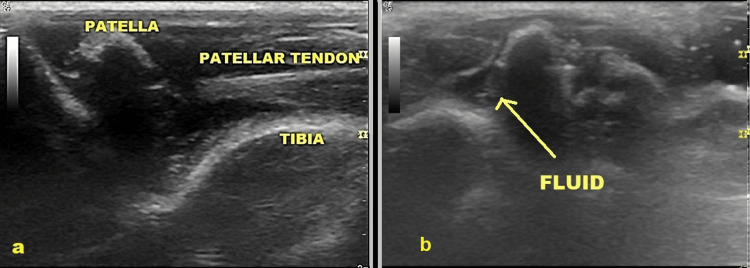
Ultrasound (US)-guided knee injection A: rat knee anatomy on ultrasound before the injection: patella, patellar tendon, and tibia are recognized; B: rat knee after the intra-articular injection; joint distention and the presence of fluid are shown.

The animals were anesthetized by intraperitoneal administration of phenobarbital (35 mg/kg). Skin preparation was carried out using povidone and an alcoholic solution. Then, 0.1 ml of the substance (either lidocaine 2% or ropivacaine 7.5%) was injected into the left knee joint of the rats. An equal volume of normal saline solution was also administered to their right knee for control. Using ultrasound guidance (GE Healthcare, LOGIQ e, Wisconsin USA), a 29G insulin syringe was inserted through the patellar tendon with the animal supine and the knee flexed to 90 degrees. The successful injection was confirmed by ultrasound, namely needle placement, joint distention, and the presence of fluid in the joint (Figure 2).

Tissue samples were obtained one, seven, 21, and 60 days after the intra-articular injection. The knee joints were excised, and the soft tissues around the tibia and femur were removed, leaving the knee joints intact. Specimens were placed in 10% buffered formalin for 24 hours at room temperature. After fixation, they were placed in a decalcification solution for two weeks and then dehydrated and embedded in paraffin. 5 μm-thick sections were obtained and treated with Safranin-O histological stain. All specimens were examined by the same pathologist. The pathologist was not aware of the substance (anesthetic or placebo) or the time elapsed from the initial injection to the sample acquisition. The modified Mankin score (Table 1) was used to evaluate the morphological changes of articular cartilage. This score has been used in previous studies regarding animal cartilage [20-21].

**Table 1:** Mankin score Modified Mankin score was used in our study to evaluate morphological changes in rat cartilage.

Modified Mankin score	
Pericellular Safranin-O staining	
Normal	0
Slightly enhanced	1
Intensively enhanced	3
Background Safranin-O staining	
Normal	0
Slight increase or decrease	1
Severe increase or decrease	2
No staining	3
Arrangement of chondrocytes	
Normal	0
Appearance of clustering	1
Hypocellularity	2
Cartilage structure	
Normal	0
Fibrillation in the superficial layer	1
Fibrillation beyond the superficial layer	2
Missing articular cartilages	3

A non-parametric Kruskal-Wallis test was chosen for statistical analysis of the resulting scores, with a significance level set at p<0.05. Post hoc analysis would follow in the case of statistically significant differences.

## Results

**Figure 3:**
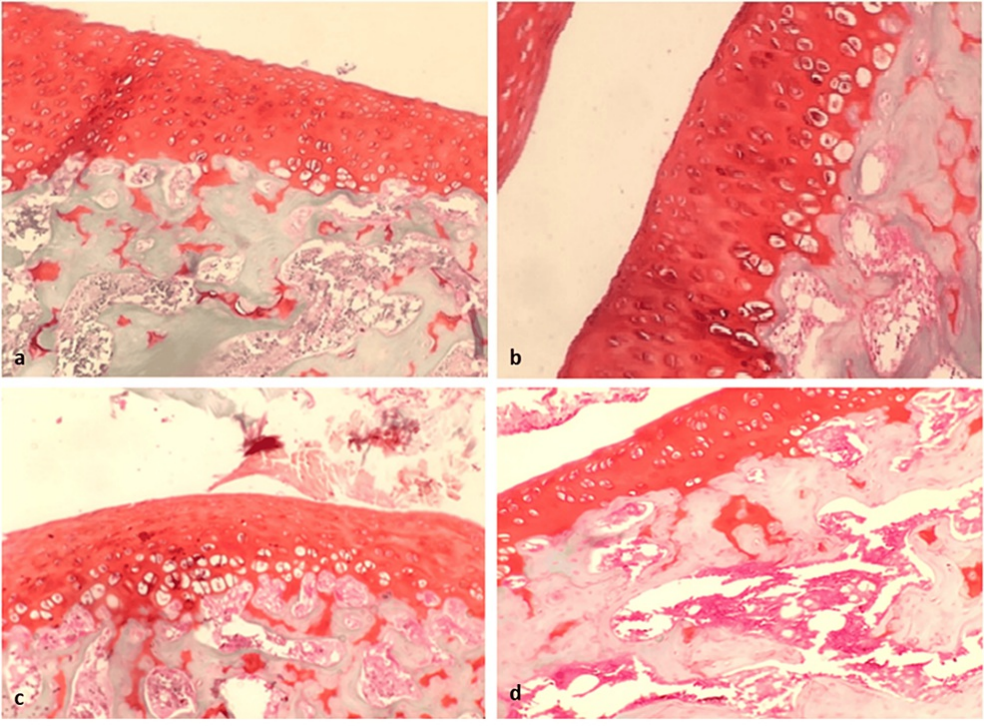
Histopathological evaluation A: Safranin-O staining of left rat knee cartilage (lidocaine); B: right knee (control); C. Safranin-O staining of left knee cartilage (ropivacaine); D: right knee (control).

All specimens were free of hematomas. Macroscopic examination did not reveal any remarkable pathological lesions. Knee cartilage sections were stained with Safranin-O and examined under a light-brightfield microscope (Figure 3). The modified Mankin score (Table 1) was used for the histopathological evaluation and grading of the articular cartilage degeneration for each individual sample. Thorough histological analysis revealed normal chondrocyte shape and arrangement in the lidocaine, ropivacaine, and placebo groups. Similarly, the extracellular matrix was not affected, and no abnormalities were detected after being stained with Safranin-O.

Consequently, the modified Mankin score was zero for all tissue sections examined, so no statistical analysis was performed. Hence, we accept the initial hypothesis that the intra-articular administration of either ropivacaine or lidocaine did not induce histopathological alteration in the rat cartilage when compared to controls. The time elapsed since the initial injection did not affect the cartilage structure either.

## Discussion

The main finding of our study is that there were no histological changes in the articular cartilage of the rats from the use of local anesthetics. This is in agreement with other similar studies. Procaine shows no significant differences in the rat’s articular cartilage when compared to the intra-articular injection of normal saline [14]. Iwasaki et al. reached the same conclusion when they tested bupivacaine in healthy and osteoarthritic rat knees [13]. Cell viability and density, as well as the Osteoarthritis Research Society International (OARSI) score, seemed to be unaffected by the infusion of bupivacaine in both osteoarthritic and healthy rat knees. On the other hand, there are studies reporting histological alterations in cartilage or synovium after intra-articular anesthetic administration in vivo. The most common finding is neutrophil infiltration on the first day after the injection [14,22]. Inflammatory responses may be observed during the fifth and tenth days after the injection [8,22-23]. The inflammatory response is measured using the Mankin or OARSI score. These scores, however, may, in some instances, not reveal the severity of cartilage damage. Indeed, Chu et al. injected bupivacaine into rat knees and examined them six months after the initial injection [21]. Through macroscopic and histological examination, the cartilage appeared intact, and histological scores showed no significant differences when compared to placebo. Nevertheless, the cartilage cell density was halved when measured by quantitative histological analysis methods six months after the injection. The mean time from continuous local anesthetic infusion to chondrolysis manifestation is approximately eight to 13.5 months. [6,24]. It is assumed that one day for the adult rat is equivalent to 34.8 human days [25]. The time interval we studied (two months) could roughly be considered equal to 70 months, or 5.8 years of human life. However, this assumption is an oversimplification, and more time may be needed to detect cartilage damage [21].

Since no cartilage damage was observed for both substances, lidocaine and ropivacaine could be considered equally safe. Nonetheless, this is not in agreement with previous studies conducted mainly in vitro. When tested in vitro, ropivacaine seems to be the least toxic among the other anesthetics [12]. On the contrary, lidocaine toxicity has been proven to be toxic to cartilage in cell cultures [10,26]. The intact cartilage matrix provides protection against local anesthetic-induced damage [11]. Local anesthetic toxicity may be enhanced when they are combined with steroids [27].

It is difficult to conclude that the intra-articular injection of local anesthetics in humans is safe, based on the findings of a single animal study. However, an extrapolation of the dose between rats and humans can provide useful data for further research. In this study, we injected a dose that was equivalent to the usual dose used in arthroscopic procedures under local anesthesia. We administered a volume of 0.1 ml. The majority of previous studies injected a fluid volume ranging between 0.1 and 0.25 ml [8,13,21-23,28]. These volumes (0.1-0.2 ml) reach the capacity of the rat knee and seem to be the reason for being selected for infusion in previous studies. An accurate extrapolation of the dose administered to the rats into a human equivalent dose may require complex calculations. The dose scaling of different species for intra-articular drugs does not follow the same rules as intravenous administration [26]. The differences in knee surface area have to be taken into consideration [15,29]. Since the rat knee joint has a 200 times smaller surface area compared to the human knee joint [29], we could hypothesize that the equivalent dose for the human would be equal to a volume of 20 ml of either lidocaine 2% or ropivacaine 7.5%. Arthroscopic procedures under local anesthesia require a minimum intra-articular administration of 10-14 ml of lidocaine 2% [16]. Ropivacaine is also administered for post-operative pain management in volumes equal to 10 ml [17]. The volume injected in this study is equivalent to the injection of 20 ml of local anesthetics in humans, thus being considered adequate for the purposes of the study.

Considering that no toxic effects were shown as a result of local anesthetic action, it was important to confirm the proper intra-articular injection of the anesthetic. Through US guidance, we verified needle placement inside the joint and joint distension after the successful solution infusion (Figure 2). To our knowledge, this is the first study utilizing US-guided infusion to ensure the accurate administration of the liquid into the joint.

The basic limitation of this study is that we evaluated the toxicity of local anesthetics based solely on histological methods. Quantitative methods could have been more sensitive in detecting differences in cell density or matrix destruction. In addition, the time interval studied may not have been adequate for cartilage lesions to develop. Moreover, healthy rat knees may be resistant to toxic effects from local anesthetics compared to injured cartilage [11]. Small sample sizes in animal studies may decrease sensitivity. Further studies, considering these factors, are desirable.

Our study did not reveal any significant macroscopic and histological differences between the intra-articular infusion of the local anesthetics compared to placebo. The dosage was extrapolated to an equivalent dose capable of inducing local anesthesia in a human knee. The accuracy of the infusion was verified by means of US assistance. We concluded that a single dose of local anesthetic administered intra-articularly does not induce any significant adverse effects in the rat cartilage when examined under a light microscope, even two months after the initial injection.

## Conclusions

A single intra-articular injection of local anesthetics did not induce any damage to the cartilage histomorphology or structure. The findings of this study imply that a single intra-articular dose of lidocaine or ropivacaine in humans may not cause any toxic effect on the cartilage in the short term. Dose scaling of intra-articular substance administration between different species is complicated. Differences in joint areas are used to estimate equivalent doses among species. Further research is required in order to establish the safety of local anesthetic intra-articular injections in humans.
